# Supporting and Enabling the Process of Innovation in Public Health: The Framework for Public Health Innovation

**DOI:** 10.3390/ijerph191610099

**Published:** 2022-08-16

**Authors:** Whitney R. Garney, Kelly L. Wilson, Kristen M. Garcia, Daenuka Muraleetharan, Christi H. Esquivel, Mandy N. Spadine, Sonya Panjwani, Kobi V. Ajayi

**Affiliations:** Department of Health and Kinesiology, Texas A&M University, College Station, TX 77843, USA

**Keywords:** systems thinking, innovation, program development, framework, model

## Abstract

This manuscript introduces a new framework for creating innovations in public health—the Framework for Public Health Innovation. The framework was developed through a longitudinal qualitative research study that investigated the process of creating innovative adolescent health programs. Interviews were conducted with a national sample of 26 organizations over two time points. Data collection focused on the process of innovative program development; organizational capacity; training; and technical assistance needs, successes, and barriers. The framework was developed and modified based on interview findings and expert advice; then, the final framework was validated with content experts. The framework illustrates a dynamic process of innovation that begins with dissatisfaction with the status quo, and then, illustrates three necessary components for innovation—space, process, and partnerships. Four categories of innovation, which range in complexity, are proposed: (1) creating a new component to an existing program, (2) adapting an existing program to meet new needs, (3) taking an alternative approach to addressing an existing program, and (4) reframing a health problem from a new perspective. As illustrated by a feedback loop, the resulting innovations disrupt the status quo. This model can be applied to any content area in public health and is useful for both research and practitioners.

## 1. Introduction

Over the past 60 years, researchers and practitioners in the social sciences have spent considerable time and resources studying innovation. One of the most frequently cited social science theories is Diffusion of Innovations by Everett Rogers [1]. Rogers describes innovation as an “idea, practice or object that is perceived as new by an individual or unit of adoption” [1]. The theory examines the diffusion of new technologies, ideas, and other forms of innovation into practice. Rogers identified factors that affect the rate at which new ideas are diffused through a social system, including innovation characteristics, characteristics of the social system, communication channels, and time.

Innovation is also examined across other disciplines, and has gained significant attention as private businesses have begun to focus on research and development. In industry, the Science Push framework was created to understand innovation as it relates to demand and environmental factors [2,3]. Over time, innovation frameworks have expanded from linear processes to circular or interactives ones, such as systems-based frameworks of innovation, which emphasize connections within and outside organizations [2]. System-based network approaches to innovation encourage collaboration among entities to diversify resources and reduce the cost of innovation [4]. Through this, organizations have learned that innovation cannot efficiently occur in silos [5,6].

Further research on translating innovation into practice has been conducted within the social sciences by scientists such as Dr. Abe Wandersman. The Interactive Systems Framework for Dissemination and Implementation (ISF) details three major systems pertaining to innovation translation: the Prevention Synthesis and Translation System, the Prevention Support System, and the Prevention Delivery System, which make up a heuristic framework [7]. The ISF illustrates how to build an organizational and innovation-specific capacity to implement new products or processes in healthcare settings. It also shows how innovation is embedded within a larger context of policy and research that influences how innovation can be adopted and replicated.

Existing innovation frameworks and models provide thought-provoking and practical applications for public health problems; however, these frameworks are not specific to the field of public health, and, therefore, have conflicting assumptions and goals. In the private sector, the underlying goals of innovation frameworks emphasize net gains instead of social good, which is a priority in public health. Furthermore, the amount of resources available to facilitate innovation processes across sectors varies greatly. In a for-profit setting, funds for research and development are common and often have tax and other benefits [8]. Many public health organizations rely on grant funds or donations to implement and sustain activities. Innovation is a luxury not often afforded to organizations working to meet people’s basic needs.

Furthermore, public health innovations must consider health disparities to advance priority areas. Health disparities are complex, and the root causes are often intertwined with interpersonal, social, and political structures; therefore, a systems approach is needed to understand and address the ecological determinants of public health problems [9]. Public health innovations should be developed through multidisciplinary and community-based collaborations, and should involve iteration to ensure that newly developed innovations are effective and accepted by the intended target population [10].

Lastly, existing frameworks most closely linked to public health priorities, such as the ISF, focus on how to apply or adopt innovation into practice; they do not describe the process of creating innovations. Therefore, this paper draws from other disciplines and five years of research in innovative public health program development to introduce a new public health approach for creating innovations: the Framework for Public Health Innovation.

### Enabling and Supporting Innovation: The iTP3 Project

The Innovative Teenage Pregnancy Prevention Project (iTP3) was developed by (BLINDED) University, and funded as a Tier 2A grantee by the Office of Population Affairs (OPA) from 2015 to 2020 [11]. The purpose of iTP3 was to develop a portfolio of innovative programs to improve adolescent health. To accomplish this, iTP3 worked with 26 organizations across the United States to develop innovative adolescent health programs.

## 2. Materials and Methods

Through the iTP3 project, this study used longitudinal qualitative research design to investigate the process of creating innovations within adolescent health programming [12]. Qualitative data were conducted at multiple time points, with personnel from 26 unique organizations as they developed innovative public health programs.

### 2.1. Longitudinal Data Collection

Three cohorts of organizations (*n* = 26) were funded to develop innovative adolescent health programs. Each organization nominated 1 to 2 staff members, directly involved in their program development activities, to participate in pre- and post-interviews. Interviewees represented the project lead, as well as support staff. Interview questions were used to understand the organization’s processes of innovative program development; capacity; training; and technical assistance needs, successes, and barriers. Interviews were conducted annually by Master’s-level, trained members of the iTP3 team [12]. The questions changed slightly between cohorts to best assess capacity and progress, given the changes in iTP3. Based on their progress, some organizations were funded for multiple years, and others for one year. Fifty-five total interviews were completed. The (AUTHOR) Institutional Review Board approved all data collection and analysis before the study.

### 2.2. Analysis

The evaluators conducted a thematic analysis using an open coding scheme with emergent codes for each round of interviews [13]. Four coders analyzed the data at each time point. The qualitative analysis began with coders reviewing each transcript and highlighting the data to break it into smaller coded segments. The coders then compared the segments and assigned themes, followed by integrating and interpreting the findings [14,15]. Batches of data were analyzed at two time points—in 2017, after the first cohort of organizations; and in 2019, after the third cohort of organizations [16].

The data analysis generated key themes at each time point. The researchers compared themes across time points to identify nuances in the data. Data were presented to the iTP3 project team for continuous quality improvement and model development.

### 2.3. Model Development

The iTP3 evaluation team created the Framework for Public Health Innovations using key themes from the first round of interviews in 2017. The ISF informed the original structure of the model; however, constructs were included that came from the thematic analysis.

The iTP3 executive committee operationalized the various aspects of the framework, and graphically illustrated them using a feedback approach, which was a key theme of the data representing iteration. The model was then discussed with outside experts, including Dr. Abe Wandersman and the project’s internal innovation advisory committee. In 2018, the iTP3 executive committee reviewed the framework to OPA and the Power to Decide, an OPA grantee focused on innovation development in technology. Lastly, modifications were made after the third cohort of interview data was analyzed in 2019, to incorporate additional insights on innovation development from organizations.

### 2.4. Validation

In January 2020, the iTP3 executive committee convened a group of content experts to validate the framework. Experts were recruited to represent people who would use this model in practice and research. Experts included representatives from public health governmental agencies; researchers in the field of adolescent health, innovation, and program development; and practitioners working on program development and implementation (*n* = 8).

The validation activities included a visual walk through of the framework, where terms and processes were operationalized. Then, experts were asked for feedback on each construct and process as presented. The validation meeting was auto-recorded for note-taking purposes. The final modifications were made based on expert feedback. Lastly, a graphic designer updated the visualization of the framework to ensure it adequately captured the theoretical and operational intent.

## 3. Results

**Figure 1:**
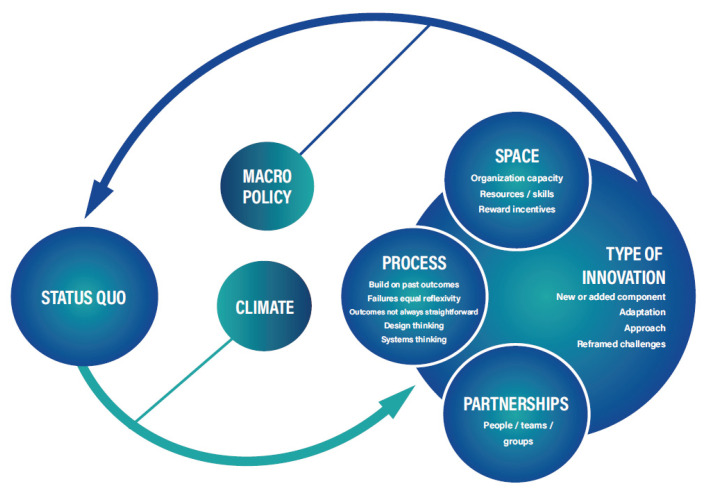
The Framework for Public Health Innovation.

A graphic representation of the Framework for Public Health Innovation is provided in Figure 1. The framework describes a developmental process in a fluid environment shaped by policy and climate. It is a feedback loop to show that innovation is both an outcome and starting point. Each construct represents key themes from the qualitative analysis. The organization, linkages, and graphics were informed by iTP3 team members and content experts.

### 3.1. Dissatisfaction with the Status Quo

To understand how organizations create innovations, it is important to capture the context of development. Similar to a new product, process, or program, an innovation is developed within an existing condition. The degree to which innovation will be accepted or adopted is based on historical circumstances and disruption. In order to represent this context, our framework shows the “status quo.” The status quo is the prologue to innovation. It is the current state of the field, and includes both climate and macro policy [17]. In “Structure of Scientific Revolutions,” Thomas Kuhn proposes that scientific paradigms are replaced based on a staged process of emergence, normal science, crisis, and revolution [18]. The Kuhnian ideas of normal science and crisis are useful for understanding the junction at which an innovation can be introduced.

Two things must be present for the potential of innovation to occur. First, the public must be dissatisfied with current circumstances. The status quo is the current state of normal science, in which practitioners and scientists use a common understanding to address problems [18]. Widespread belief in an approach and shared knowledge of truth supports the actions of a collective. However, when new discoveries emerge that cannot be explained or addressed by normal science (the status quo), disruption occurs. In these instances, scientists disagree about the right approach, and needs remain unmet; therefore, a crisis, as Kuhn describes, emerges. The situation represents dissatisfaction with the status quo in this framework [18]. Dissatisfaction is necessary for innovation because it propels the desire for newness, meaning more unsatisfied people results in greater potential for radical change [19].

Second, the climate is crucial for innovation. Climate represents social and cultural norms surrounding the health topic [7]. The public support and political climate vary based on the health topic of focus. Finally, “macro policy” informs the status quo because it can limit or propel potential innovation through funding, support, and sanctions [7].

### 3.2. Space, Process, and Partnerships

The second component of the framework describes three essential aspects of innovation—space, process, and partnerships.

#### 3.2.1. Space

The space to be innovative is necessary to cultivate innovation, but not sufficient on its own. It represents organizational buy-in for innovation, such as supportive leadership, incentives for employees to pursue innovation, as well as resources and skills to develop and respond to innovation [20]. Innovation cannot occur without appropriate space [21].

#### 3.2.2. Process

The process represents an iterative approach to innovation that allows for failure and reflexivity [22]. This component acknowledges that outcomes in innovation are not always straightforward [23]. For instance, when a new program is developed and tested, but the results are not positive, this can be a success. Instead of investing time and resources in that program, the process allows iteration and learning from failure, and then moves on. Based on experiences in iTP3, the framework supports two specific processes which can drive innovation—design thinking and systems thinking. Both design and systems thinking are processes themselves, but when viewed within this larger framework, they ensure key elements such as ecology and human-centeredness are incorporated into health innovation [24,25]. New skills and professional development, such as learning design or systems thinking, are important to innovation [26,27].

##### Systems Thinking

The word “system” refers to a set of interconnected things [28]. This could be a group of people, or departments within an organization. In public health, a system often refers to organizations; however, this is only one way to define a system [29]. Authors use a broader definition when referring to systems thinking in this paper, which is a set of interconnected things, so that they yield their pattern of behavior over time [28]. Systems thinking views “the whole as more than just the sum of its parts” [30]. It recognizes the interconnectedness of factors, and how feedback on these factors produces health behaviors [31]. We live in a world where complex, interconnected issues require us to go beyond traditional, linear thinking [32]. Take the example of homeless youth. Homelessness is an issue resulting from various factors, including environmental circumstances, financial resources, social norms, access to services, and more. It cannot be distilled into a single cause or set of factors [33]. Therefore, systems thinking can be used to better understand this system problem [34]. Systems thinking is used to understand relationships and patterns, and then use those patterns to shift a system’s behavior [35]. We must embrace complexity and think broadly [25].

Because of the complexity of systems problems, frequently, people disagree on the best solution. Some scholars use the terms “cloud” and “clock” to describe systems and non-systems problems [36]. The term “cloud” is assigned to a systems problem. Karl Popper, the philosopher, said, “clouds are intended to represent systems which, like gases, are highly irregular, disorderly, and more or less unpredictable;” on the other hand, clocks represent “systems which are regular, orderly, and highly predictable in their behavior” [36]. Clock problems can be solved with precise changes to object A, seen in object B. The processes for addressing them are more straightforward. When we encounter a cloud problem, such as teenage pregnancy, we cannot use a linear approach to solve it; therefore, systems thinking is useful.

##### Design Thinking

Design thinking is a development process that allows for tolerance, visualization, quick iteration, and prototyping [37]. Design thinking was originally applied within the business sector, but it is a process that can apply to any discipline, including social innovation [16,23,38,39,40,41,42]. Design thinking enables participants to engage in strategies to develop solutions with people or communities in mind by moving away from conventional problem-solving [23,43] collaboratively. The iterative process includes five key phases, not occurring in a sequential or ordered fashion: (1) develop empathy, (2) define needs and problem(s), (3) ideate and brainstorm, (4) build prototypes, and (5) test prototypes [44]. Projects often move back and forth between the phases multiple times as ideas are refined, and new avenues are considered [23,44].

Thousands of design thinking strategies exist. Through these interactive techniques, participants first establish empathy with people/users (human-centered design) and communities (community-centered design) [23]. Empathy allows participants to develop a deep understanding of their target populations’ needs, and identify problems or challenges to be addressed [38,40]. Upon understanding the root problem, participants engage in converging and diverging strategies to iteratively brainstorm, develop, and refine ideas while incorporating user feedback [40]. This process continues a rapid cycle to maintain momentum and develop innovations efficiently [23,45]. As promising solutions are developed, they are presented to end-users for feedback, allowing developers to refine the innovation while still at the drawing board, before investing extensive resources.

Some techniques are similar to the qualitative research methods used in public health, including observations, interviews, contextual inquiry, and immersion [38,46,47]. Through these strategies, researchers and developers can use the insights and experiences of the end-user as invaluable expertise regarding the core problem [48,49]. Relative to social services, design thinking is now increasingly used in public health to meet societal needs through innovative programs and practices [43]. When using human-centered design or community-centered design, teams are using techniques to develop solutions with people or the community in mind.

#### 3.2.3. Partnerships

The last process component of the framework is partnerships. This includes people, teams, or groups who engage in the process of innovation [50,51]. As previously described, it implies they reside within a space to be innovative, and includes the people who will engage in the systems and design processes. Partnerships represent individuals with the capacity for innovation, as well as working practices that ensure teamwork throughout development [51,52]. Because innovation is not routinely engaged within public health, support mechanisms and social connectedness are essential.

In the iTP3 project, partnerships were found in project teams, collaborations across entities with shared goals, and supportive peers who also worked on innovative program development. All partners were useful to the process of developing innovation, but in various capacities. The project teams were responsible for collaborating with one another to conduct design activities and prototype programs. The collaborators at different organizations were essential in securing funding, providing feedback, and gaining access to target populations. Lastly, peers also working on innovative program development were useful for morale and providing feedback on shared experiences.

### 3.3. Type of Innovation

The iTP3 project revealed variations in the types of innovation in public health. Early evaluation results defined innovation in terms of: (1) the target population being addressed, (2) a program’s mechanism of delivery, and (3) the program development approach [16]. However, in the later years of the iTP3 project, four variations in the type of innovation emerged. These types of innovation increased in complexity, which corresponded to a disruption of the status quo.

#### 3.3.1. New Component

The first type of innovation is a new component to an existing health product, program, or process. This is the simplest type of innovation because it adds to existing practices to meet a need rather than replacing it. For example, a new component might be adding social media to an existing sex education program in response to user needs or changes in technology or societal norms [53]. It does not radically alter the existing program or product; rather, it makes it more relevant.

#### 3.3.2. Adaptation

The second type of innovation is adaptation. Adaptations represent a modified way of doing something, such as adapting an existing evidence-based program (EBP) for a new target population [54]. The fundamental theory of change stays the same for adaptation to occur, but things such as language or setting are changed. Frequently, we see adaptations to meet the needs of different cultural groups or genders in teenage pregnancy prevention programming.

#### 3.3.3. Approach

The third type of innovation is the approach. The approach is more drastic because it represents a new way of doing something. An example of a new approach is a trauma-informed approach to sexuality education, or the switch from abstinence-only to comprehensive sexuality education [55,56]. Frequently, a new approach means a new guiding theory and requires a change in terminology. Therefore, it causes a great deal of disruption because it represents something completely different instead of adapting or adding a new component to an existing program or product.

#### 3.3.4. Reframing

The last and most radical type of innovation is reframing the problem or challenge completely [57]. How we understand a public health challenge directs the potential for intervention [58]. This type of innovation is similar to a paradigm shift in Kuhn’s Scientific Revolution because it implies a new understanding of a health issue, new methods to address the issue, and a new way of understanding success [59]. An example of a reframed challenge is the HIV and AIDS epidemic. When AIDS was first discovered, scare tactics and radical strategies, such as banning the entry of HIV-positive people into the US, were used [60]. Public health programs increased awareness and provided basic information regarding symptoms. As science progressed and public opinion shifted, programs using scare tactics and discrimination were replaced with anti-stigma campaigns focusing on antiretroviral treatment [61,62]. In this example, the public health challenge was reframed from dying of AIDS to living with HIV.

#### 3.3.5. Feedback

The type of innovation that emerges from this framework informs the status quo and aligns with the early health technology assessment (HTA). In essence, early HTA reflects systems and design thinking in that it takes into consideration the viewpoints of critical stakeholders (e.g., the hospital, patient, assessor, the medical device industry, and the policy-makers) when developing new technology for optimal health outcomes [63]. The status quo is constantly shifting as innovation emerges and is translated into practice. The feedback loop connecting the circles represents this movement.

## 4. Discussion

The Framework for Public Health Innovation builds on Roger’s work, and seeks to expand our knowledge of the diffusion of innovations that transcend beyond the proven methods and frameworks of the past [1]. The framework focuses on the components and processes needed to develop innovations specific to public health. 

The model was developed using adolescent health programming as an opportunity to study the process of innovative program development; however, the model can be applied to all topics within public health. The model can also be used by public health practitioners, researchers, and funding agencies as they develop new ways to address existing health issues.

### 4.1. Public Support

The Framework for Public Health Innovation can be applied to a variety of public health priority areas. As depicted in the model, all innovation starts with a status quo and existing context, including climate and policy. One reason that a model specific to the field of public health is critical is because of the public nature of the discipline. The majority of public health work is publicly funded through government agencies or foundations; therefore, it is impossible to separate public support from innovation. If the public does not support the topic of innovation, it is very difficult to progress. Model users should be aware of the importance of public support, and consider the policy and climate surrounding health topics before entering into innovative development activities. 

### 4.2. Design Process and Systems Focus

The Framework for Public Health Innovation describes two specific processes to push innovation—systems thinking and design thinking. The iTP3 project successfully used these processes to facilitate the development of innovative adolescent health programs [11]. There are several approaches to design, including design boot camps or design sprints, which can be helpful [64]. The authors encourage people interested in design to pursue existing resources available online. 

The framework emphasizes a systems perspective via its visual depiction and components. It uses a feedback loop to show that innovation is both an outcome and starting point. The feedback loop illustrates that innovation is a process in a fluid environment shaped by policy and climate. As a component of systems thinking, feedback loops show that the actions taken by a person or group of people eventually affect that same person or group [63,65]. They also illustrate dynamic properties in systems, and show how a problem is created and maintained over time [66]. In fact, innovation and sustainability require that issues are analyzed holistically, reflecting the health and social systems (e.g., social, ethical, and legal climate) that predict health innovations and programs, and by extension health outcomes [67].

Systems thinking is a way to understand relationships and patterns within systems, and then use those patterns to shift a system’s behavior [35]. Systems thinking recognizes the relationship between a system’s structure and behavior [68]. A paramount distinction between system-level and individual-level theory is that systems theory emphasizes how determinants are interconnected and embedded within a larger context rather than focusing on linear causality. The proposed framework includes systems thinking as a process component because it is impossible to distill complex public health issues down to a chain of single factors. Systems thinking acknowledges feedback among determinants, the creation of emergent properties, and the complexity to understand how a problem occurs and its context [28].

### 4.3. Need for Target Population Feedback

Public health innovation must be framed around target population needs and holistic approaches [39,67]. Through the iTP3 project, the researchers found that programs developed by some organizations in the first cohort targeted underserved populations, but were not using youth feedback directly in the development process. Though this was not wrong, the programs that emerged were not different from existing teenage pregnancy prevention programs. In the second and third cohort of organizations, the developers began to use design and systems processes [16]. As systems thinking and design thinking were utilized, a critical shift occurred, and the resulting innovations varied greatly from existing programs, and were designed directly to meet the needs of potential users.

### 4.4. Aptitude for Innovation

Though potentially controversial, the iTP3 team found that another important consideration is that not all people or teams can develop innovative approaches [16]. This does not mean that all people and teams cannot be innovative, because anyone can adopt an innovation. However, developing innovations requires a great deal of flexibility and the ability to trust a process, rather than entering a project with an outcome in mind, and then working towards that metric. Public health needs people and teams that can develop innovations, as well as those who adopt or implement standardized approaches; therefore, neither trait is right nor wrong. However, it is important to acknowledge that creating innovations is a learned skill. Tools exist to identify individuals’ innovative characteristics, such as Innovation 360 or the Innovation Strengths Preference Indicator [69,70]. These can help identify people who are best suited to pursue innovation development; however, through the iTP3 project experience, we believe the best way to identify innovative teams is through experience. Innovation is iterative, so people become more familiar with the process as they participate. Through these interactions, people develop the capacity for innovation.

## 5. Conclusions

The Framework for Public Health Innovation builds on past social science literature, and uses primary research to identify a process to develop innovations within public health. This framework is useful to researchers and practitioners who want to challenge the current state of science, and introduce new programs and products to meet stakeholder needs.

## Data Availability

Not applicable.
